# Learning Semantics of Gestural Instructions for Human-Robot Collaboration

**DOI:** 10.3389/fnbot.2018.00007

**Published:** 2018-03-19

**Authors:** Dadhichi Shukla, Özgür Erkent, Justus Piater

**Affiliations:** Intelligent and Interactive Systems, Department of Computer Science, University of Innsbruck, Innsbruck, Austria

**Keywords:** human-robot collaboration, proactive learning, gesture understanding, intention prediction, user study

## Abstract

Designed to work safely alongside humans, collaborative robots need to be capable partners in human-robot teams. Besides having key capabilities like detecting gestures, recognizing objects, grasping them, and handing them over, these robots need to seamlessly adapt their behavior for efficient human-robot collaboration. In this context we present the fast, supervised Proactive Incremental Learning (PIL) framework for learning associations between human hand gestures and the intended robotic manipulation actions. With the *proactive* aspect, the robot is competent to predict the human's intent and perform an action without waiting for an instruction. The *incremental* aspect enables the robot to learn associations on the fly while performing a task. It is a probabilistic, statistically-driven approach. As a proof of concept, we focus on a table assembly task where the robot assists its human partner. We investigate how the accuracy of gesture detection affects the number of interactions required to complete the task. We also conducted a human-robot interaction study with non-roboticist users comparing a *proactive* with a *reactive* robot that waits for instructions.

## 1. Introduction

Human teams are exceptionally good at conducting collaborative tasks, from apparently trivial tasks like moving furniture to complex tasks like playing a symphony. Humans can communicate task-relevant information by verbal as well as nonverbal channels such as gestures. Presumably this is one of the reasons why working in teams is seen to be beneficial. Humans can also predict the intent of the partner by observing the current state of a task. We need collaborative robots with suchlike capabilities for effective human-robot teams.

**Figure 1:**
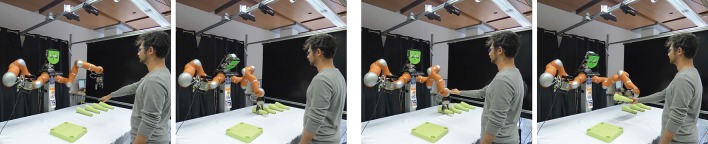
Robin (our robot) assists the user in the assembly of a table. The user performs gestures like pointing and give me to which Robin reacts by grasping the object and handing it over, respectively.

Thanks to advances in the field of robot control and computer vision, it has been possible to develop frameworks for human-robot teams to perform collaborative tasks. Consider a domestic scenario like table assembly as illustrated in Figure 1. The overall task is composed of sub-tasks like detecting a gesture, identifying the targeted object, grasping the object, handing the object to the user, or placing the object within reach of the user.

In such tasks, different users will have different preferences for the sequence of actions expected from the robot. Certainly, a robot taking these preferences into account and having an operational flexibility would be favored for natural human-robot interaction (HRI). We go one step further and endow the robot with proactive behavior—to predict the intent of the user and act accordingly–based on observing the past and the current state of the task. To establish a robot system with these abilities we present the fast, supervised Proactive Incremental Learning (PIL) framework. This work expands upon our previous version of the framework (Shukla et al, 2017a).

The PIL framework is designed to learn the associations between human hand gestures and the robot's manipulation actions. The robot can perform a manipulation action given that it is aware of three main attributes: the state of the human, the state of the objects to manipulate, and its own state. The state of the human is the instruction command given by the user; here, it is a static hand gesture performed by the user. The state of an object is given by whether it is in the robot's hand or not. The robot's own state is given by the manipulation action it just finished performing. These attributes together define the state of the system. The next manipulation action which the robot will perform is selected based on the probability of an action given the state of the system.

### 1.1. Motivation and contribution

Our framework is motivated by joint intention theory for sequential actions, proposed by Cohen and Levesque (1991) to design a collaborative framework. One of the ways the authors describe *collaboration* is the *joint stepwise execution*, where a team intends to do a sequential action by having a mutual belief about the state of the goal at each step thereafter embarking on the remainder of a task. Interesting works (Chao et al., 2010; Myagmarjav and Sridharan, 2015; Cruz et al., 2016) have shown how a human or an oracle can teach a robot the associations between instructions and actions. However, these frameworks include a prior training phase as part of the pipeline to perform a task. On the other hand, the PIL framework is free of prior learning of the associations. Rather, the gesture-action associations are learnt incrementally on the fly, an advantage in terms of total interaction time. In our previous work (Shukla et al, 2017b) we showed benefits of proactive learning over a pre-trained system in overall interaction time.

The PIL framework is a probabilistic, statistically driven approach. It comprises of two main aspects:

1. Its *proactive* nature enables the robot to predict the intent of the user. The proactive behavior becomes active once the associations begin to consolidate, i.e., after some number of interactions. Consequently, based on the learnt probabilities of the gesture-action associations, the robot can decide on the most likely action.
2. Its *incremental* learning paradigm gives the user the freedom to establish gesture-action associations at will. Training and testing are not two distinct phases in the PIL framework. Instead, both are active till the system reaches the goal. Additionally, the PIL framework comprises of an *action anticipation* module that enables the robot to anticipate its next action. The action anticipation module is active when the gesture-action associations are not known.

We use the gaze of the robot to establish common ground between the robot and the user. Since our robot cannot speak and does not use a screen to communicate its intentions, it uses its head (or gaze) to complete the communication cycle. Studies have shown how gaze can serve to indicate the readiness of the robot (Bee et al., 2009) or to signal planned actions followed by an action (Huang et al., 2015). In the work by Fischer et al. (2015), the authors explore the effects of social gaze in a collaborative toolbox assembly scenario with naive users. Their analyses show that a robot with active gaze engages people faster compared to the one without it. Our framework incorporates gaze in ways found to be effective in these studies. We use the gaze of the robot for two main purposes, (1) to establish mutual belief, i.e., to indicate to the user the action it is about to take, and (2) to indicate when it is ready for the next instruction. For example, the robot will look at its hand if it is going to either close it or open it, or it will look at the object if it is going to reach for it.

**Figure 2:**
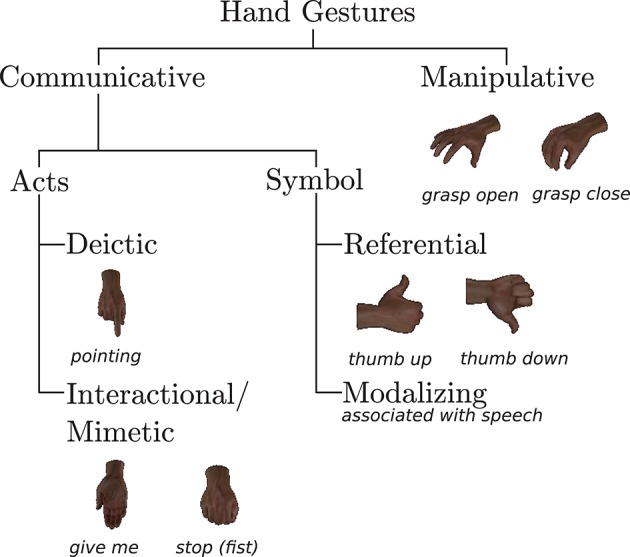
Taxonomy of hand gestures of the IMHG dataset.

The human hand is naturally deformable and varies in shape, size, and color, which makes hand pose estimation a challenging field of research. The table assembly task in this work takes place in close proximity; therefore it is likely that the robot (due to a limited field of view) can only see the user's hand. Thus it becomes irrelevant to observe full-body gestures. Owing to these reasons, we restrict ourselves to use static hand gestures from the Innsbruck Multi-view Hand Gestures (IMHG) dataset^1^ (Shukla et al., 2016) to instruct the robot. They were designed based on our previous HRI study (Jensen et al., 2015) conducted with participants having no experience in interacting with the robot. They are closely related to the semantic content of verbal language. These gestures can be categorized based on Quek (1995)'s taxonomy as shown in Figure 2.

Furthermore, hand gestures inherently provide spatial information of the user's hand (Shukla et al., 2015). Gesture detection systems are prone to misclassification due to various factors including lighting conditions, gesture appearance ambiguities, etc. The PIL framework enables the robot to proactively correct misclassified gestures. We simulated a HRI scenario of assembling a table to study the effect of the accuracy of gesture detection in performing the assembly.

To present realistic findings of the PIL framework, we conducted a human-robot interaction study in the real robot environment with non-roboticist participants. The HRI scenario designed for the study is as shown in Figure 1, where the user has to assemble a table and the robot's role is to assist the user. Two robot behaviors were implemented on the real robot, *reactive* and *proactive*. In the reactive behavior, the robot learns associations incrementally but it cannot predict the user's intent. It always waits for an instruction from the user before taking any action. In the proactive behavior, the robot behaves using the PIL framework.

In summary, the contributions of this paper are

1. the PIL framework with a novel probabilistic action anticipation module,
2. the study of the effect of the rate of gesture detection on the overall interaction, and
3. a human-robot interaction study with naive users comparing *reactive* and *proactive* behaviors of the robot.

### 1.2. Related work

In the field of human-robot collaboration, numerous studies (Bandera et al., 2012; Rozo et al., 2016) demonstrated the advantage of active, human-in-the-loop interaction. The framework proposed by Lenz et al. (2008) allows the joint action of humans and robots for an assembly task. Their system can anticipate human behavior based on the learnt sequence, ensuring smooth collaboration. An active-learning architecture proposed by Myagmarjav and Sridharan (2015) can be trained with limited knowledge of the task. It enables the robot to ask task-relevant questions to acquire information from the user. The work by Chao et al. (2010) belongs to the same category. Nevertheless, a pertinent difference with these methods is that PIL does not require an explicit training phase.

Based on Q-learning (Watkins and Dayan, 1992), Thomaz and Breazeal (2006) introduced the Interactive Reinforcement Learning (IRL) method. In this approach, the user is able to provide positive and negative rewards during training in response to the robot's manipulation action. The authors demonstrated that human-generated reward can be fast compared to classical reinforcement learning. Along these lines, in the work by Suay and Chernova (2011) the user provides guidance signals to constrain the robot's exploration toward a limited set of actions. Here, the user provides feedback for every action. Najar et al. (2016) proposed a similar IRL method to learn the meaning of the guidance signals by using evaluative feedback instead of task rewards. Recent work by Rozo et al. (2016) is built on the same concepts. In contrast to our approach, the IRL methods do not incorporate proactive robot behavior.

Our framework is along the lines of the work discussed below in the sense that it provides the robot with proactive behavior in a collaboration task. Huang and Mutlu (2016) presented an *anticipatory control* method that enables the robot to proactively perform a pick-and-place task based on anticipated actions of their human partners. Their anticipation module is trained using eye-tracking glasses which track the gaze of the user. The authors showed that anticipatory control responded to the user significantly faster than a reactive control method that does not anticipate the user's intent. Hawkins et al. (2014) constructed a probabilistic graphical model to anticipate human action. In their work, users wore brightly-colored surgical gloves while giving instructions to the robot. Caccavale and Finzi (2017)'s attentional behavior-based system uses a hierarchical architecture. It recognizes human activities and intentions in a simulated environment to pick and place objects. All three approaches require prior learning of the task to model the anticipatory behavior. Contrary to these approaches, we use hand gestures which are a natural, unencumbered, non-contact, and prop-free mode of interaction in the real robot environment.

In our previous implementation of the framework (Shukla et al, 2017a), the robot randomly performed an action if the association between the state of the system and the action was unknown. After each action the robot receives feedback (positive or negative) from the user. If the action was given a negative feedback then it randomly chooses another action. One contribution of this work is a probabilistic *action anticipation* module that ranks candidate actions. The rank of an action is decided based on the probability of the action given the three attributes of the state of the system. Details of action anticipation module are discussed in section 3.2. The action anticipation module helps to sort the sequence of the manipulation actions instead of choosing them randomly, therefore speeding-up the task.

## 2. Human-robot collaboration scenario

### 2.1. Human gestures and robot actions

**Figure 3:**
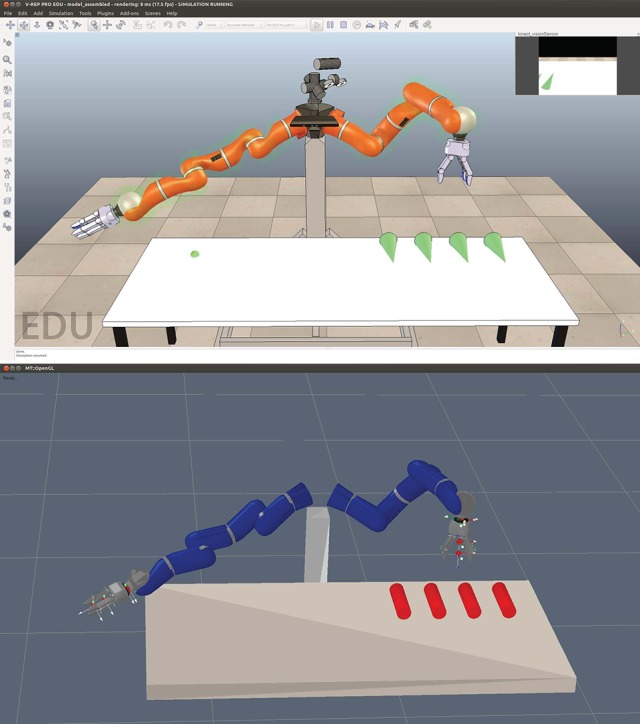
**(Top)** Simulated scene of Robin in V-REP; **(Bottom)** KOMO motion planner to plan and execute manipulation actions.

The HRI scenario is set up with “Robin” (our robot)—an anthropomorphic configuration of two KUKA light-weight robot arms, two Schunk SDH-2 hands, and a KIT robotic head (Asfour et al., 2008)—in a simulated and a real environment. Only the left arm of Robin is active during the task. To simulate the HRI environment we use the V-REP robot simulator^2^. We use the KOMO motion planner (Toussaint, 2014) to plan and execute robot arm movements. The simulation and the motion planner are illustrated in Figure 3. The goal of the robot is to assist its human partner by delivering legs of the table.

Let *G* = {pointing, give me, grasp, release} be the set of four instructional gestures performed by the user. The intended semantics of these gestures are as follows: pointing indicates an object or a position in the workspace, give me means to bring the object toward the user, grasp means to close its hand, release means to open its hand. Nonetheless, the PIL framework provides the flexibility to learn these semantics as per the user's choice. Since Robin learns the associations based on the user's feedback, let *F* = {ok, ¬ok} be the set of known feedback signals. The signals ok, ¬ok are given by performing thumb up and thumb down gestures, respectively. The HRI study by Jensen et al. (2015) shows that for an appropriate robot action often no feedback or rarely positive feedback is given. On the other hand, users consistently gave negative feedback for unexpected actions. Therefore, in addition to thumb up, we also consider *no feedback* as ok. The appearances of gestures in *G* and in *F* are known to Robin's gesture detection system prior to the task, however not their meanings. We use our probabilistic appearance-based pose estimation (PAPE) method (Erkent et al., 2016; Shukla et al., 2016) to detect hand gestures. It is based on probabilistic representations of objects and scenes by Teney and Piater (2014).

Let *A* = {open, close, object, human, location} be the set of five actions available to Robin: open is to open its own hand, close is to close its own hand, object is to move its hand above a given object, human is to move its hand toward the user, and location is to move its hand to a given location indicated by the user in the workspace. In our previous work (Shukla et al, 2017a) we discussed benefits of the robot's gaze; therefore all actions in *A* are preceded by a directed gaze. Let *E* = {hand, object, palm, position, face} be the set of five gaze directions. Robin uses the first four gaze directions to communicate to the user the action it is going to perform next. Gaze directed at its own hand indicates that action open or close will be performed next. It will direct its gaze at the object to indicate action object. Robin will gaze position to indicate location, whereas gazing at the user's give me gesture is represented by palm. Lastly, Robin can look at the user's face indicating that it is ready for the next gesture. These associations between gaze directions in *E* and the actions in *A* are known to the user prior to the task.

The state of the object to be manipulated mainly depends on the state of Robin's hand. The two states of its hand representing whether or not it is currently holding an object are defined by the set *H* = {occupied, free}.

### 2.2. Task execution

The state *s* of the system consists of three attributes at any time step *t*. It is defined as *s*_*t*_ = 〈*g*_*t*_, *a*_*t*_, *h*_*t*_〉, where *g*_*t*_ ∈ *G* is the gesture detected by the robot, *a*_*t*_ ∈ *A* is the action the robot performed at step *t*, and *h*_*t*_ ∈ *H* is the state of the robot's hand. Let *R* = {*g*_*t*_, *a*_*t*_, *h*_*t*_} be the set of these attributes. At each step *t* of the assembly, the robot records three entities, the state *s*_*t*_ of the system, the action *a*_*t*+1_ ∈ *A* the robot will perform next, and the feedback signal *f*_*t*+1_ ∈ *F* given by the human partner after the action. As mentioned earlier, each action *a*_*t*+1_ is preceded by a gaze movement *e*_*t*+1_ ∈ *E*. The predicted gesture is given by *g*_*t*+1_ ∈ *G*.

Consider the sequence of septuples as shown in Table 1, describing handover of four legs of the table. Each block delimited by dotted lines is one gesture-action association (e.g., steps 1–4), and each block delimited by solid lines is one handover (e.g., steps 1–10). Since *e* = 〈face〉 is not associated with any action in *A*, it is excluded from the sequence of interactions in Table 1. However, Robin looks at the user's face after action *a*_*t*+1_. For the purpose of explanation let us consider that gestures are detected accurately. Initially, Robin is at a home position with its hand open, and the four legs of the table are kept within its reachable workspace.

**Table 1:** An example trace of the interactions leading to four handovers while learning gesture-action associations.

***t***	***g*_*t*_**	***a*_*t*_**	***h*_*t*_**	***e*_*t*+1_**	***f*_*t*+1_**	***a*_*t*+1_**	***g*_*t*+1_**
1	pointing	open	free	position	¬ok	location	
2		open	free	palm	¬ok	human	
3		open	free	hand	¬ok	close	
4		open	free	object	ok	object	
5	grasp	object	free	palm	¬ok	human	
6		object	free	position	¬ok	location	
7		object	free	hand	ok	close	
8	give me	close	occupied	palm	ok	human	
9	release	human	occupied	position	¬ok	location	
10		human	occupied	hand	ok	open	
11	pointing	open	free	object	ok	object	grasp
12	grasp	object	free	hand	ok	close	give me
13	pointing	close	occupied	palm	¬ok	human	
14		close	occupied	object	¬ok	object	
15		close	occupied	hand	¬ok	open	
16		close	occupied	position	ok	location	
17	release	location	occupied	hand	ok	open	
				palm		human	
				object		object	
18	pointing	open	free	object	ok	object	grasp
19	pointing	object	free	object	ok	object	
				hand		close	
				position		location	
				palm		human	
20	grasp	object	free	hand	ok	close	give me
21	pointing	close	occupied	position	ok	location	release
22	release	location	occupied	hand	ok	open	pointing
23	pointing	open	free	object	ok	object	grasp
24	grasp	object	free	hand	ok	close	pointing
25	pointing	close	occupied	position	ok	location	release
26	give me	location	occupied	hand	¬ok	open	
27				palm	ok	human	
				object		object	
28	release	human	occupied	hand	ok	open	pointing

At step *t* = 1, the user points at one of the legs of the table. Robin's vision system detects the hand gesture as pointing. Since no associations have been recorded so far, the probability of choosing an action is uniformly distributed over all the actions from *A*. At this point, Robin cannot anticipate action *a*_1+1_; therefore it randomly selects one of the actions, here location. The gaze paired with action *a*_1+1_ = 〈location〉 is *e*_1+1_ = 〈position〉, i.e., to look at a location in the workspace. The user observes the robot looking at a location indicating it wants to move its empty hand there. Knowing that the position gaze in a free hand state indicates an imminent location action, here the user immediately reacts by giving ¬ok feedback because location is not the desired action. The actions colored in red in column *a*_*t*+1_ are not executed. Similarly, ¬ok is also given upon the next, randomly-selected palm gaze, which is paired with action human. Finally, at step *t* = 4 the user gives ok, the object gaze signaling that the robot is about to perform the intended object action. It is to be noted that action open is not indicated since the robot just performed action *a*_*t*_ = 〈open〉 and *h*_*t*_ = 〈free〉. The PIL framework will eliminate redundant actions.

Next, the user instructs Robin to grasp the object by performing the grasp gesture. A similar procedure as above follows and Robin learns the association between the gesture grasp and the action close. At some point, the robot will have learnt that it has to execute the object action given that it had performed action open, the state of its hand is free, and the state of the user is pointing. Consequently, at *t* = 11 the gesture prediction is activated. It rightly predicts *g*_11+1_ = 〈grasp〉 based on prior knowledge. Though based on learnt probabilities, predicting *g*_12+1_ = 〈give me〉 is not incorrect; it fails because the user decides to deviate from the learnt sequence. The correct and incorrect gesture predictions are highlighted in green and red in column *g*_*t*+1_, respectively.

The gaze states highlighted in blue in column *e*_*t*+1_ indicate when Robin's action anticipation module is active. If action *a*_*t*+1_ is unknown given the state *s*_*t*_ of the system then Robin uses the acquired knowledge to anticipate an action given the attributes in *R*, i.e., *g*_*t*_, *a*_*t*_, *h*_*t*_. It ranks candidate actions by computing the conditional probabilities of an action given these attributes. Initially, Robin fails at *t* = 13 since the acquired information is insufficient. However, at *t* = 17 and *t* = 19 the actions are ranked such that the anticipated action is in coherence with the user's desired action. The action location is not one of the ranked actions. The action performed by the robot *a*_17_ = 〈location〉; therefore it is redundant to indicate the same action. At *t* = 20 the predicted gesture could be give me or pointing since both have happened only once. In such cases, when gestures have equal probabilities the PIL framework randomly chooses one of the gestures. At *t* = 25 Robin performs action *a*_25+1_ = 〈location〉 and predicts *g*_25+1_ = 〈release〉. It ranks open first at *t* = 26 indicating it with gaze *e*_26+1_ = 〈hand〉. These predictions are in line with the interaction so far, but then the user decides to change the course of the sequence. It can be seen at *t* = 26 that the user instructs the robot to hand over the grasped leg instead of delivering it to the target pointed at. Robin then hands over the leg at *t* = 27. This procedure continues till all legs have been handed to the user.

## 3. Proactive incremental learning

The PIL framework is designed to reach the final state of the task minimizing both the number of user-demonstrated gestures as well as the number of robot actions. Let us consider the collaborative table assembly scenario from section 2 with *N* the number of legs Robin has to hand over to the user. The PIL framework consists of three modules: (1) incremental gesture-action association, (2) action anticipation, and (3) proactive gesture prediction and correction.

### 3.1. Incremental gesture-action association

The underlying assumption of the PIL framework is that the user wants to complete the task in a minimum number of interactions. The robot hence learns *P*(*a*_*t*+1_|*s*_*t*_) denoting the probability of an action *a*_*t*+1_ to be executed given the system's current state *s*_*t*_. At first, the probabilities are distributed uniformly among all the manipulation actions of the robot. The robot uses the feedback from the user to incrementally learn these probabilities at every step *t*. The action *a*_*t*+1_ to perform given the state *s*_*t*_ of the system is selected as

(1) $$\begin{array}{l} a_{t+1}=\underset{a}{\text{argmax }}P\left(a|s_{t}\right). \end{array}$$

As mentioned previously, each action is paired with a gaze movement that precedes the actual action. The user then provides feedback ok or ¬ok based on whether the robot has indicated an appropriate action to advance toward the goal. If the user signals ok then the robot goes ahead executing the action; otherwise it will indicate another action using its gaze.

Let T be a 4-D table storing, at each step *t*, the frequency of ok feedback signals given to an interaction, where the interaction comprises of the state of the system and the next action. The frequencies in T then form the basis of frequentist probability estimates *P*(*a*|*s*) of the associations. As the task progresses, the frequencies in T for interactions (*s*_*t*_, *a*_*t*+1_) are updated. We can thus compute the probability of an action *a* given the state *s* of the system as

(2) $$\begin{array}{l} P\left(a|s\right)=\frac{T\left(s,a\right)}{\overset{\left|A\right|}{\underset{i=1}{\sum }}T\left(s,a_{i}\right)}. \end{array}$$

The frequencies in T are update based on the user's feedback. If the user signals ok for an indicated action *a*_*t*+1_ then cell T(*s*_*t*_, *a*_*t*+1_) is incremented as

(3) $$\begin{array}{l} T\left(s_{t},a_{t+1}\right)\leftarrow T\left(s_{t},a_{t+1}\right)+1. \end{array}$$

If the user rejects the indicated action by ¬ok feedback then the frequencies of all state-action associations other than the currently-indicated action are incremented by 1. The frequencies of all possible actions $a_{t+1}^{\times }\in A$ except *a*_*t*+1_ are updated as

(4) $$\begin{array}{l} T\left(s_{t},a_{t+1}^{\times }\right)\leftarrow T\left(s_{t},a_{t+1}^{\times }\right)+1,a_{t+1}^{\times }\neq a_{t+1}. \end{array}$$

We refer to this as a *complement feedback* technique.

Previous methods (Suay and Chernova, 2011; Najar et al., 2016) give either −1 or 0 for ¬ok feedback. If we update Equation 3 with −1 for ¬ok then frequencies in T can attain negative values, making it difficult to derive probability estimates *P*(*a*|*s*) from them. Moreover, the frequencies in T will be updated without actually representing the occurrence of the state-action (*s*_*t*_, *a*_*t*+1_) pairs. Similarly, if zero is assigned to ¬ok feedback then frequencies in T will remain unchanged. Thus, a ¬ok would be equivalent to no feedback at all, discarding a useful piece of information from the user. In contrast, the complement feedback technique maintains the probabilistic nature of the PIL framework by taking both positive and negative user feedback into account while guaranteeing positive and consistent probability estimates. For example, at *t* = 1 in Table 1, *s*_1_ = 〈pointing, open, free〉, *e*_1+1_ = 〈position〉, and *a*_1+1_ = 〈location〉, the robot receives ¬ok feedback. As a consequence, T(*s*_1_, location) is not incremented whereas T(*s*_1_, human), T(*s*_1_, close), T(*s*_1_, open), and T(*s*_1_, object) are incremented by 1.

### 3.2. Action anticipation

The key advancement of PIL compared to its precursor (Shukla et al, 2017a) is to anticipate the most likely robot actions if gesture-action associations are unknown. This results in a substantial reduction in the number of steps necessary to complete a task. The PIL framework computes a probability distribution over all possible manipulation actions and ranks them from *most anticipated* to *least anticipated*. To anticipate the next action *a*_*t*+1_, the framework computes two conditional probabilities for each action in *A* given the attributes of the state *s*_*t*_ = 〈*g*_*t*_, *a*_*t*_, *h*_*t*_〉 of the system, namely the conditional probability

(5) $$\begin{array}{l} P\left(a_{t+1}|r_{i}\right)=\frac{P\left(a_{t+1},r_{i}\right)}{P\left(r_{i}\right)} \end{array}$$

of an action given only one attribute *r*_*i*_ ∈ *R*, and the conditional probability

(6) $$\begin{array}{l} P\left(a_{t+1}|r_{i},r_{j}\right)=\frac{P\left(a_{t+1},r_{i},r_{j}\right)}{P\left(r_{i},r_{j}\right)},\;i\neq j \end{array}$$

of an action given two attributes *r*_*i*_, *r*_*j*_ ∈ *R* simultaneously. Despite the three attributes of *s*, at most only two attributes are used to estimate the *most anticipated* action. If all three attributes of *s* are used then Equation (6) will be equivalent to Equation (2). In such a case, to learn a new state-action association an action will be randomly chosen since all actions will have equal probabilities.

Initially, the joint probabilities *P*(*a*_*t*+1_, *r*_*i*_) and *P*(*a*_*t*+1_, *r*_*i*_, *r*_*j*_) are unknown and taken to be uniform. They are estimated by counting observations in T indexed by attributes of *s*_*t*_ and *a*_*t*+1_. Using the conditional probabilities in Equations (5, 6), we compute the score *q* for each candidate action *a*_*t*+1_ ∈ *A* as

(7) $$\begin{array}{l} q_{a_{t+1}}=\underset{i,j}{\max}(P\left(a_{t+1}|r_{i}\right),P\left(a_{t+1}|r_{i},r_{j}\right)),\;i\neq j. \end{array}$$

The action that maximizes score *q* is ranked as the most likely anticipated action with the remaining actions ranked in descending order of *q*.

### 3.3. Proactive gesture prediction and correction

The particularity of the PIL framework is minimizing the effort of the user, i.e., the number of gestures performed by the user, thereupon it lessens the overall interaction time. The proactive gesture prediction enables the robot to decide on action *a*_*t*+2_ associated with *g*_*t*+1_. The prediction module is active once the framework has recorded the frequency of ok signals given to interactions in T. In addition to T, the framework also stores the history of interactions. The robot refers to the history of interactions to compute the probability of the next gesture *g*_*t*+1_ given the current state *s*_*t*_ of the system and the associated action *a*_*t*+1_, i.e., *P*(*g*_*t*+1_|*s*_*t*_, *a*_*t*+1_). The gesture attaining the highest probability *P*(*g*_*t*+1_|*s*_*t*_, *a*_*t*+1_) is selected as the predicted gesture *g*_*t*+1_. However, if the user decides to diverge from the learnt sequence then a different gesture can be performed after the execution of *a*_*t*+1_.

Poor accuracy of a gesture detection system generally causes misclassification of an instruction gesture and as a result, it can evoke an invalid state of the system. For example, pointing is misclassified as grasp when the robot is holding an object. Invalidity of a state may be detected in two main ways: (1) A gesture is detected with a low confidence score irrespective of the state of the system; therefore it is discarded. (2) A misclassified gesture is incompatible with the state of the system irrespective of the confidence score. We address this problem with a methodology similar to that described for *gesture prediction*. The gesture correction module too is activated after associations are recorded in T. If the detected gesture *g*_*t*_ yields an invalid state then no *a*_*t*+1_ is selected. At this point, the system checks for all the associations followed by (*s*_*t*−1_, *a*_*t*_) with ok feedback.

The framework computes the *P*(*g*_*t*_|*s*_*t*−1_, *a*_*t*_). The gesture with the highest probability is chosen as the *corrected* gesture. Based on the updated state *s*_*t*_, the robot then performs gaze *e*_*t*+1_ which is paired with *a*_*t*+1_. The user always has the freedom to provide ¬ok feedback and discard robot's selection. The robot then selects the next best action and indicates it using gaze. If none of the previously-learnt manipulation actions are acceptable by the user then the robot explores other state-action associations with ¬ok feedback.

Consider again the sequence of interactions in Table 1. At *t* = 22, if *g*_22_ = 〈release〉 is misclassified as grasp, it would trigger an invalid state. It is not possible for the robot to grasp an object when the state of the hand is occupied. At this point, the system then decides that gesture correction is necessary. Based on the learnt probabilities the most likely gesture to occur after *s*_22−1_ = 〈pointing, close, occupied〉 when *a*_22_ = 〈location〉 is release. The detected gesture is corrected from grasp to release, and the robot proceeds to perform the action open.

## 4. Experiments

We conducted three sets of quantitative experiments to evaluate the PIL framework. The experiments involved assembling tables, where the robot assists by handing legs of the tables over to the user. In other words, the table assembly task is used to examine the efficacy of the framework to learn the gesture-action associations. The first two experiments are aimed at evaluating performance of the PIL framework in comparison to the state of the art. The objective of these experiments is to optimize the effort required to perform table assemblies. This includes learning of the gesture-action associations while performing the assembly task, until the task has been completed. Learning is always active because the user has the freedom to change the sequence of task execution or semantics of gestures during the task. We compare the following methods:

1. A reactive system and IRL methods (Suay and Chernova, 2011; Najar et al., 2016), capable of learning gesture-action associations, but lacking *gesture predicting* and *action anticipating* capabilities;
2. PIL1 (Shukla et al, 2017a), capable of learning gesture-action associations as well as predicting the user's next gesture and proactively correcting misclassified gestures;
3. PIL2 (the proposed framework), an extension of PIL1 that adds action anticipation capabilities.

The first experiment was performed in a simulated environment as shown in Figure 3. We evaluated two objective metrics, the number of hand gestures performed by the user and the number of the robot's manipulation actions. The second experiment used the real robot to measure three evaluation metrics, namely, interaction effort, neglect tolerance, and robot attention demand, for human-robot interaction as proposed by Olsen and Goodrich (2003). Lastly, we conducted a within-subject HRI study with naive users to obtain subjective responses for comparing reactive and proactive robot behaviors. These responses show us how the two robot behaviors were perceived by the participants in regards to their performance during the table assembly. Like the first experiment we also evaluated the two objective metrics from the video recordings of the HRI study to compare them with the subjective responses of the users.

### 4.1. Simulated environment

We simulated the table assembly scenario described in section 2 by iteratively assembling three tables. In total the robot has to hand over 12 table legs either into the user's palm or to a pointed location in the workspace. We repeated this interaction 20 times. The necessary gestures and feedback are provided via an external user interface. We simulated three detection rates *d* = {0.6, 0.8, 1.0} to evaluate the effect of accuracy of the gesture detection system on the number of hand gestures and the number of robot actions needed to complete the task. We quantitatively compared the proposed PIL framework (PIL2) with four existing approaches: two interactive reinforcement learning (IRL) methods, IRL1 (Suay and Chernova, 2011) and IRL2 (Najar et al., 2016), our previous version of PIL (PIL1) (Shukla et al, 2017a), and reactive robot behavior.

The IRL methods have shown promising results in learning tasks involving human feedback. The two IRL implementations were adapted for the table assembly scenario. The learning rate α and the discount factor γ for IRL1 and IRL2 are set as mentioned by the authors to α = 0.3, γ = 0.75 and α = 0.3, γ = 0.0, respectively. The authors of IRL2 argue that γ = 0.0 is more suitable for learning from human feedback. It allows a task to be divided into a sequence of single-step tasks. Note though that γ = 0.0 would take into account only immediate rewards, therefore rendering the reinforcement learning aspect incongruous for overall task learning. Additionally, we also present results of both IRL methods at a higher learning rate α = 0.9. In our previous work (Shukla et al, 2017b) we showed how the robot's gaze facilitates communication and speeds up completion of the task. Therefore, for a fair comparison we incorporate gaze into both IRL methods. Also, *no feedback* was considered as ok feedback.

As mentioned before, our previous PIL implementation (PIL1) does not include the *action anticipation* module. However, it can proactively predict the intent of the user, i.e., gesture, and perform an action if the gesture-action association is known. On the other hand, the reactive robot learns the associations incrementally but waits for an instructional gesture at every step after each action. The reactive robot can neither perform gesture prediction or correction, nor can it anticipate the next action.

**Figure 4:**
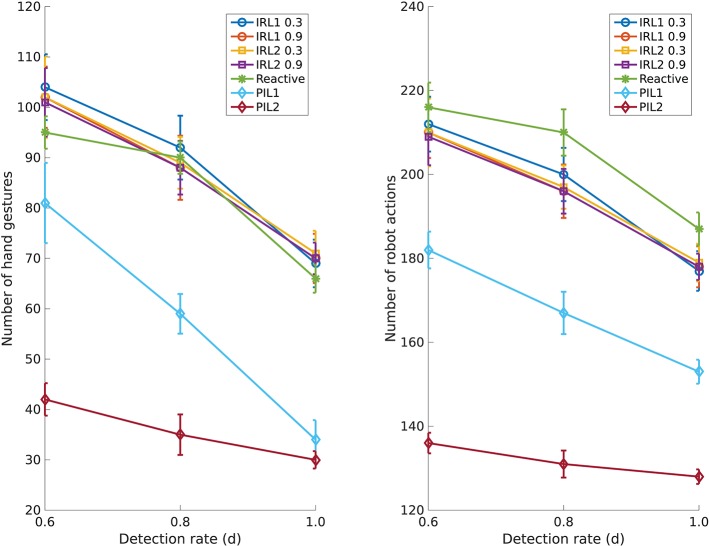
Effect of the gesture detection rate on the table assembly task. **(Left)** Number of hand gestures needed at various detection rates; **(Right)** Number of robot actions needed at various detection rates.

Figure 4 shows comparisons with respect to the number of hand gestures performed by the user and the number of robot actions. The number of hand gestures by the user is the sum of the number of instructional gestures from *G* and the number of feedback gestures from *F*. The number of robot actions is the sum of the number of manipulation actions from *A* and the number of gaze movements from *E*. A general observation from the plots is that as the detection rate increases the effort of the user and the number of robot actions reduces for all the approaches. The IRL methods are reactive in nature, i.e., they require an instructional gesture at every step. As a consequence, they neither predict the next gesture nor anticipate the next action. Therefore they need more gestures as well as robot actions. Interestingly, the factor by which the effort reduces as the detection rate increases is significantly smaller for PIL2 as compared to the other methods. Clearly, PIL2 capitalizes on its action anticipation module resulting in fast human-robot interaction, even at low gesture detection rates.

Figure 4 shows that numbers for both aforementioned criteria reduce significantly with the PIL1 framework and even more with the proposed PIL2 framework. Since PIL2 is able to predict the user's next gesture and it is able to anticipate the next robot action, it frees the user from the effort of performing an instructional gesture or a feedback gesture. Likewise anticipating robot actions reduces the number of robot's gaze movements since each action is preceded by a gaze. The proactive behavior allows the robot to proceed with the task without attention from the user.

### 4.2. Real environment

The experiment with the real robot consisted of the same interaction setup as described in section 4.1. We computed three evaluation metrics, viz., Interaction Effort (IE), Neglect Tolerance (NT), and Robot Attention Demand (RAD). These are all time-based metrics that attempt to maximize the speed of performance, minimize mistakes, and measure the autonomy of the robot.

*Interaction effort* is the amount of time the user has to invest in interacting with the robot. We compute IE as the cumulative sum of the time taken by the robot to detect hand gestures, both instruction gestures from *G* and feedback gestures from *F*. The user needs to maintain the gesture until it has been detected. Once the gesture is detected the robot acknowledges this with an immediate gaze suggesting the next action. It takes 1.5–2.5 s for the PAPE method to detect one instance of the hand gestures of the IMHG dataset (Shukla et al., 2016). The goal of the system is to reduce IE and lead the robot toward proactive behavior. *Neglect tolerance* represents tasks which a robot can perform without human supervision. The NT was computed as the cumulative sum of the time taken by the robot to perform various sub-tasks, namely planning the trajectory of the robot arm using KOMO, executing the trajectory, opening and closing its hand, and executing its gaze movements. Both IE and NT are measured in seconds.

*Robot attention demand* is the relation between IE and NT, given by

(8) $$\begin{array}{l} \text{RAD}=\frac{\text{IE}}{\text{IE}+\text{NT}}. \end{array}$$

RAD is a unitless quantity that represents the effort that the user expends in interacting with the robot relative to the total robot time. A good human-robot interaction system tries to minimize the RAD value. A low RAD indicates that the user can focus on other tasks besides interacting with the robot. Since RAD mainly measures the effort that the user has to invest in the interaction we compare the proposed PIL2 approach with PIL1 and the reactive robot behavior.

**Figure 5:**
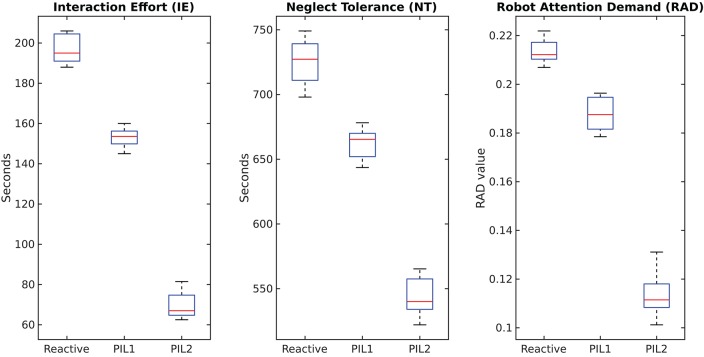
Comparison of HRI evaluation metrics for Reactive behavior, PIL1, and PIL2.

We evaluate our method by assembling three tables with the real robot and measuring execution times. We repeat the experiment five times, the results of which are presented in Figure 5. The IE and RAD values clearly show that the user has more free time while interacting using the PIL2 framework compared to the other two approaches (PIL1 and reactive). In the proposed scenario of table assembly, a high NT does not necessarily suggest a better HRI. The value of NT can be increased by slowing down the speed of the robot, yet we would like to finish assembling the table in the shortest time possible. Although NT is higher for reactive behavior and for PIL1 than for PIL2, it does not imply a better experience since the overall interaction time increases due to a higher number of robot actions.

### 4.3. User study

Like for the previous two experiments we chose the table assembly scenario also for our user study. As participants perceived Robin's arm movements as too slow (Jensen et al., 2017), to avoid boredom during the experiment, we merged action close with action object, and action open with actions human and location. Before starting the experiment, participants were informed about the overall task, available gestures, Robin's workspace, its actions, and its field of view. To the participants were also explained the associations between Robin's gaze movements and actions. In particular, three gaze movements were explained. Robin would perform: (1) gaze object prior to reaching and grasping an object, (2) gaze palm before delivering the grasped object into the participant's hand (action human), and (3) gaze position before placing the grasped object at a targeted location in the workspace (location). The interactions were recorded after receiving a written consent of the participants. Two questionnaires were asked to be completed, a demographic questionnaire before the experiment and a post-experiment feedback questionnaire.

A total of 22 participants (14 female, 8 male) took part in our study, with a mean age of 30.3 and a standard deviation of 7.0 years. Participants were from non-robotic fields of studies like law, medicine, linguistics, biology, physics, or economics. Their experience with a robot was limited to having seen one at an exhibition or in movies; therefore, no actual experience in collaborating with a robot was collected previously. All participants were asked to assemble two tables, where Robin interacted once using the reactive behavior and once using the proactive behavior. They were divided into two groups, based on which behavior they encountered first. Group 1 first interacted with the reactive robot and subsequently with the proactive robot, whereas group 2 first interacted with the proactive robot followed by the reactive robot. No information about the two distinct behaviors of the robot was given to the participants except that the second assembly restarts the task from scratch without any memory of the previous assembly.

We test three hypotheses in this study:

**H1:** The proactive robot will be perceived as an efficient partner to work with.
**H2:** The reactive robot will be deemed as an engaging robot throughout the interaction.
**H3:** Participants will consider the proactive robot as a quick learner compared to the reactive robot.

We analyse two objective criteria similar to the experiment in section 4.1, the number of hand gestures performed by the user and the number of robot actions in both assemblies. The results comparing the two groups are shown in Table 2, where μ is the mean and σ is the standard deviation. In both groups some participants did not take into account the gaze of the robot, more so during the proactive behavior. They performed gestures even when the robot was busy with an action, e.g., grasping or moving its arm, i.e., during a robot gaze other than face. Therefore the standard deviation is higher for proactive behavior than for reactive behavior. This shows that, perhaps for some participants it was straightforward to adapt to the reactive robot than to the proactive robot. Nevertheless, the proactive robot requires fewer gestures and actions to finish the task.

**Table 2:** Objective results comparing proactive and reactive behaviors of the robot.

		# **Hand gestures**		# **Robot actions**	
**Group**	**Behavior**	**μ**	**σ**	**μ**	**σ**
1	Reactive	9.4	0.5	28.5	1.9
	Proactive	5.8	1.9	23.5	1.3
2	Reactive	9.2	0.4	28.0	1.8
	Proactive	6.5	2.1	23.8	1.5

During the user study we observed great variation in the behavior of participants when they performed a gesture. For example, some gestures were inadvertently shown outside Robin's field-of-view; some gestures were shown for a very short period of time resulting in no detection; some participants first gave verbal instructions before resorting to hand gestures. Due to such nonuniformity in behavior of the participants we do not compute time-based objective metrics like IE, NT, and RAD for naive users.

The goal of this user study was to compare three principal aspects of both behaviors: (1) workload on the participants, (2) productivity of the robot, and (3) interactivity of the robot. Since the table assembly task does not require intense physical effort, the *workload* is enquired as the effort the participants had to put into performing gestures, and the time taken to complete the assembly. The *productivity* of the robot is how the participants perceived the learning capability of the robot, and how efficient it would be in performing a repetitive task in a factory-like-setting to help a human-worker. Finally, the *interactivity* of the robot is how the participants rated the robot's skill to communicate its intentions about next actions, and to what degree users felt engaged during the interaction. The following questions from the post-experiment questionnaire provide us subjective information to compare both behaviors:

**Q1:** In which assembly did you feel the most effort?
**Q2:** Which assembly did you feel the most time consuming?
**Q3:** How would you rate the task learning capability of the robot during both assemblies?
**Q4:** Which behavior of the robot would you prefer in a factory setting to assemble 30 tables?
**Q5:** How would you rate the robot's skill to communicate its intentions about its next action?
**Q6:** How engaging was the robot in each assembly?

**Figure 6:**
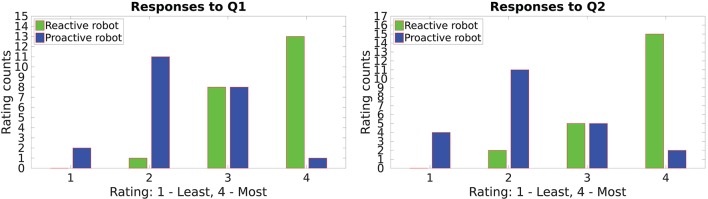
Workload on the participants while doing the task with the proactive and the reactive behavior.

**Figure 7:**
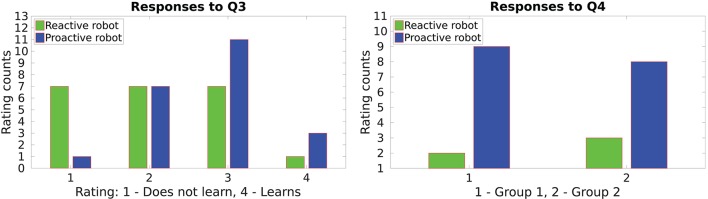
Productivity of the robot in the proactive and the reactive behavior.

**Figure 8:**
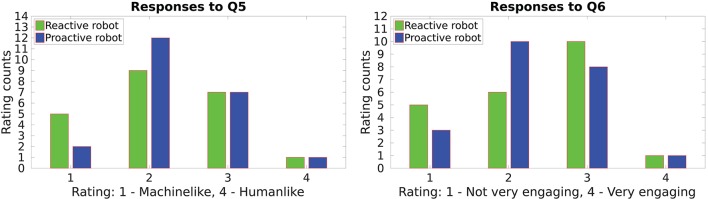
Interactivity of the robot in the proactive and the reactive behavior.

Figures 6–**8** show accumulated responses from participants of both groups.

The responses to questions **Q1** and **Q2** are summarized in Figure 6. The users were asked to rate both, table assembly 1 and table assembly 2. It can be seen that most users felt the workload was reduced when the robot predicted their gestures and acted accordingly. Most users rated the proactive behavior for *least effort and time* while the reactive behavior got a majority for *most effort and time*. Overall ratings are in favor of the proactive robot. These responses concur with the objective evaluation shown in Table 2.

The proactive framework mainly focuses on learning the gesture-action associations; consequently, the robot also learns the sequences of actions. Since the proactive robot is capable of acting on its own, most of the participants in both groups perceived it as a better learner than the reactive robot. This can be observed from the responses to questions **Q3** and **Q4**, shown in Figure 7 which enquire the productivity of the robot. Evidently, in both groups the proactive behavior was preferred in an imaginary factory-like-setting when doing a repetitive task. Some participants however favored the reactive behavior which might imply that they would like to have full control over when the robot performs an action.

The responses to **Q5** and **Q6** which investigate the interactivity of the robot are depicted in Figure 8. Despite the various benefits of using gaze as shown in previous HRI studies, the gaze of the robot was not perceived as *humanlike*—intuitive to interpret—to indicate its intention (or the next action). The responses of the participants were inclined toward *machinelike* gaze—it is not intuitive and needs additional effort to interpret—for both behaviors. Conversely, gaze proved to be an important factor in engaging the users duringe interaction. The reactive behavior emerges as an *engaging robot* as compared to the proactive behavior since the former always performs a face gaze after each manipulation action and waits for the next instruction.

In addition to the subjective responses, we asked the participants to express the experience in their own words. The comments from the participants provide further insight on how both behaviors of the robot were perceived. We quote a few comments of the participants of both groups in favor of the reactive robot and the proactive robot in Tables 3, 4, respectively. Participants of group 1 refer the reactive robot as assembly 1 and the proactive robot as assembly 2, whereas in group 2, assembly 1 and assembly 2 implies the proactive robot and the reactive robot, respectively. The comments in Table 3 indicate that although the proactive robot is efficient to perform the task, for some participants it is not a preferred choice. It can be deduced from the comments that the participants felt the reactive robot was a better choice since it gave them better control over when the robot performs an action as well as it kept them engaged during the task using the gaze face. On the other hand, the comments in Table 4 reflect that participants appreciated the proactive robot for performing the task quickly and liked its increased level of autonomy. The comments suggests that working with the proactive robot seemed more comforting than the reactive robot.

**Table 3:** Comments in favor of the reactive robot.

**GROUP 1**	
1.	“Assembly one was more fun, the robot seemed more interactive and more humanistic. The second assembly seemed more automated, however was slightly quicker.”
2.	“Assembly 1 required a lot more effort, but since the robot was more reliant on instructions, there was also more communication. In Assembly 2, the robot was much more efficient, but it felt more like he was giving me orders, since his actions were less informed by my imperatives, but my actions were more informed by his.”
**GROUP 2**	
1.	“In my opinion Assembly 1 was more efficient, but Assembly 2 felt more human. So I would prefer to work with Assembly 2.”
2.	“Assembly 1 the robot kept going without waiting for my instructions. Assembly 2 the robot was completely engaged.”

**Table 4:** Comments in favor of the proactive robot.

**GROUP 1**	
1.	“I found the robot is well responding to my indications. In general, I felt more comfortable in interacting with the robot for Assembly 2 rather than for Assembly 1.”
2.	“Assembly 1 was more difficult, since the robot seemed to take longer to process my commands whereas in assembly 2, it felt as if it was already “expecting” my commands and thus processed them faster and more accurately.”
**GROUP 2**	
1.	“There was a decisive difference and slower response with assembly 2. It was a curious experience. I did not know if I was supposed to look into the face/eyes or the camera under it to make contact with the machine.”
2.	“Hard to differentiate, I feel Assembly one was more responsive.”

These comments and the responses from post-experiment questionnaire support all the three hypotheses.

## 5. Discussion

For human-robot collaboration scenarios, both verbal and non-verbal modes of communication come with a challenge of teaching semantics of instructions to the robot. In most HRI frameworks the robots are capable of responding to a small number of pre-programmed instructions (Mavridis, 2015). By means of these instructions the user can interact with the robot to perform a task. However, with such frameworks the user is obliged to follow a strict mapping between instructions and robot actions; there is no learning. On the other hand, the main goal of the PIL framework is to give freedom to users to teach semantics of instructions as they choose. Unlike previous works (Lenz et al., 2008; Myagmarjav and Sridharan, 2015), in PIL the mapping between an instruction and a robot action materializes during the task, therefore bypassing a training phase. We demonstrated in our previous work (Shukla et al, 2017b) the advantage of learning semantics of instructions while performing the task using PIL over pre-trained associations.

As discussed earlier, we use static hand gestures as the means of instructing the robot. However, the PIL framework can be used with other modes of communication such as speech, dynamic gestures, etc. The PIL framework allows the user to incrementally teach association between a hand gesture and a robot action. Currently, the robot is aware of appearances of the instructional gestures *G* and semantics of the feedback gestures *F*. Although for PIL play to its full strength, users should be allowed to introduce new gestures during a task. One possibility could be to incorporate on-the-fly gesture learning in the PIL framework. It is a topic of active research in gesture recognition (Fanello et al., 2013; Cabrera and Wachs, 2017; Cabrera et al., 2017). Once a gesture is recorded, the user can teach its association with a robot action. In addition to learning new associations during one task, it should be possible to reuse/modify the learnt associations when performing other tasks.

Our quantitative analysis reported in section 4 demonstrates that a robot with intent prediction capabilities can reduce the user's effort and can facilitate achieving the task quickly. While a reactive robot behavior waits for an instruction from the user at every step of the task, it compels the user to engage with the robot. This was also observed in our user study where non-roboticist participants had to assemble two tables with the robot behaving either proactively or reactively. The proactive robot begins to act independently after acquiring sufficient knowledge about the task. The learning capability of the proactive robot was greatly appreciated by most participants. Although the proactive robot scores high in learning and efficiency, it comes at the cost of poor interactivity with the user.

Some practical issues arose during the user study while working with the proactive robot. Sometimes the robot proactively took the decision to deliver a leg of the table while the user was still busy attaching another leg. In an another instance, it was observed in post-study video analysis that a participant had already planned to show a gesture to the robot but had to retract since it began to execute an action. While the proactive robot focussed on efficiency and reducing the user's effort, nonetheless it was perceived as automated, unengaging behavior by some participants. Contrary to our assumption, for a small set of participants the reactive robot emerged as a preferred choice since they can control when the robot should execute an action.

While much of our work presented in this paper is focussed on robot learning gesture-action associations, we also studied how the gaze of the robot was perceived by the participants of our user study. Building on prior research (Ruesch et al., 2008; Bee et al., 2009; Fischer et al., 2015), we used the gaze of the robot to indicate the action it is about to perform and to indicate when it is ready for the next instruction. The study by Huang et al. (2015) demonstrates how a robot can facilitate a collaborative task by observing human gaze cues. However, the reverse, i.e., the human observing the robot's gaze cues, was not found to be equally effective. Unlike human-human interaction where use of gaze cues is strikingly productive, in our study the robot's gaze was found to be counter-intuitive. The subjective responses of the participants shown in Figure 8 indicate that, except for gaze face they had to invest effort into interpreting gaze movements associated with actions. To overcome such challenges, future frameworks must further exploit and experiment with gaze interaction models inspired by human-human interaction (Boucher et al., 2012). For example, the robot can alternate between the face gaze and the gaze associated with an action to draw the user's focus of attention toward the next action.

## 6. Conclusions

We proposed a fast, supervised PIL framework to learn the associations between human hand gestures and robot manipulation actions. We also introduced an action anticipation module to reduce the number of user gestures as well as the robot actions required. The results from simulated and real-world experiments show that the proposed PIL framework outperforms state-of-the-art methods. Moreover, working with the proactive robot reduces the interaction effort (IE) since it learns to predict the next gesture.

The results of our user study uncover differences as seen from a non-roboticist's perspective. In general, the participants of our user study favor collaboration with the proactive robot. Yet, a small faction did not feel comfortable with the robot making its own decisions, thus favor the reactive robot. Further, in the case of both behaviors the robot's gaze was majorly perceived as *machinelike*, i.e., not intuitive, to indicate the next action. On the other hand, the proactive behavior was perceived as an efficient choice despite lacking interactive capabilities due to increased autonomy.

Based on these results and observations we conclude that the following facets are essential to design an effective human-robot collaboration framework

1. the user should be able to teach the rostbot semantics of instructions at will,
2. the robot should act proactively to achieve the goal in fewer interactions, and
3. the user should be kept in the learning loop using means like the gaze of the robot.

The responses of the user study suggests that future collaborative frameworks must be able to switch between the proactive and the reactive behavior. For example, if the user is busy in a task and cannot receive an object then the robot should pause its action and wait until the user is free. The robot can continue once the user gives an instruction to proceed. The robot can alter its behavior during the interaction by monitoring user's gestures at all time. Moreover, the gaze of the robot requires improvement on ways to indicate intent of the robot.

## Ethics statement

The user study conducted in this work is exempt from approval by the Board of Ethical Issues, University of Innsbruck, Austria. Prior to participating in this study, each participant signed a form giving explicit consent to video recordings of their interaction with the robot and to the use of these data for research purposes and research-related publication. The informed-consent forms signed by all participants comply with the EU-FP7 guidelines on Ethics in research and international cooperation, and so do the technical and administrative measures that were put in place to ensure the protection and privacy of the personal data (name, age, gender, video recordings, etc.).

## Author contributions

All authors listed have made a substantial, direct and intellectual contribution to the work, and approved it for publication.

### Conflict of interest statement

The authors declare that the research was conducted in the absence of any commercial or financial relationships that could be construed as a potential conflict of interest.

## Footnotes

^1^https://iis.uibk.ac.at/datasets/imhg

^2^http://www.coppeliarobotics.com/
